# Platelet-rich plasma injection for the treatment of ankle osteoarthritis: a systematic review and meta-analysis

**DOI:** 10.1186/s13018-023-03828-z

**Published:** 2023-05-19

**Authors:** Sukij Laohajaroensombat, Suwimol Prusmetikul, Sasivimol Rattanasiri, Ammarin Thakkinstian, Patarawan Woratanarat

**Affiliations:** 1 Department of Orthopedics, Faculty of Medicine Ramathibodi Hospital, Mahidol University, 270 Rama VI Road, Ratchathewi, Bangkok, 10400 Thailand; 2 Department of Clinical Epidemiology and Biostatistics, Faculty of Medicine Ramathibodi Hospital, Mahidol University, Bangkok, 10400 Thailand

**Keywords:** Conservative treatment, Dosage, Function, Pain, Preparation

## Abstract

**Background:**

Platelet-rich plasma (PRP) injection for ankle osteoarthritis (OA) treatment showed contradictory results. This review was aimed to pool individual studies which assessed the efficacy of PRP for ankle OA treatment.

**Methods:**

This study was conducted following the preferred report items of systematic review and meta-analysis guideline. PubMed and Scopus were searched up to January 2023. Meta-analysis, or individual randomised controlled trial (RCT), or observational studies were included if they involved ankle OA with aged ≥ 18 years, compared before–after receiving PRP, or PRP with other treatments, and reported visual analog scale (VAS) or functional outcomes. Selection of eligible studies and data extraction were independently performed by two authors. Heterogeneity test using Cochrane *Q* test and the *I*^2^-statistic were assessed. Standardised (SMD) or unstandardised mean difference (USMD) and 95% confidence interval (CI) were estimated and pooled across studies.

**Results:**

Three studies from meta-analysis and two individual studies were included, which consisted of one RCT and four before–after studies with 184 ankle OAs and 132 PRP**.** The average age was 50.8–59.3 years, and 25–60% of PRP injected cases were male. The number of primary ankle OA was accounted to 0–100%. When compared to before treatment, PRP significantly reduced VAS and functional score at 12 weeks with pooled USMD of − 2.80, 95% CI − 3.91, − 2.68; *p* < 0.001 (*Q* = 82.91, *p* < 0.001; *I*^2^ 96.38%), and pooled SMD of 1.73, 95% CI 1.37, 2.09; *p* < 0.001 (*Q* = 4.87, *p* = 0.18; *I*^2^ 38.44%), respectively.

**Conclusion:**

PRP may beneficially improve pain and functional scores for ankle OA in a short-term period. Its magnitude of improvement seems to be similar to placebo effects from the previous RCT. A large-scale RCT with proper whole blood and PRP preparation processes is required to prove treatment effects.

*Trial registration* PROSPERO number CRD42022297503.

**Supplementary Information:**

The online version contains supplementary material available at 10.1186/s13018-023-03828-z.

## Introduction

Ankle osteoarthritis (OA) presents with progressive cartilage destruction and may affect walking, physical and functional abilities, and also quality of life. Its incidence has been systematically approximated at 3%, particularly in the young and female [1]. According to primary (idiopathic) or secondary OA (caused by trauma, infection, or inflammation), treatment goals are pain control and regaining functions via disease-modifying methods.

Various conservative managements (e.g. rest, activity modification, shoe insert, and articular injections with saline, sugar, etc.) including PRP have been widely used [2–6]. The evidence showed promising results among hyaluronic acid [3, 4] and probably PRP [6] in improving pain and foot–ankle functional scores. There were a systematic review and meta-analysis [6] which assessed PRP effects on talar osteochondral lesions [7–9] and trapeziometatarsal arthritis [10]. This review found that PRP significantly improved functional score for at least six months of follow-up with mean difference of 11.79 (95% confidence interval (CI): 5.21–18.26) when compared to controls [6]. However, these findings may be inapplicable for ankle OA. One robust randomised controlled trial has been recently compared PRP to placebo control among patients with ankle OA [11]. The results showed non-significant difference of pain and function between two groups. On the other hand, two cohorts of ankle OA [12, 13] applied PRP injection with activated processes and demonstrated significant pain reduction.

The contradictory results among diverse populations, treatments, and level of evidences bring about a gap of knowledge. Therefore, this study questioned “Does the PRP improve pain and function for ankle OA when compared to other conservative treatments?”. This systematic review and meta-analysis were conducted to pool the effect of PRP on pain and functional scores in ankle OA patients. This study hypothesised that PRP administration for ankle OA would positively alleviate pain and promote function.

## Methods

### Eligibility criteria

Eligible criteria for individual studies identified from meta-analysis were individual studies within systematic reviews and meta-analysis either RCTs, non-RCTs, cohort study, before–after study, or case series; involving ankle OA in adults aged at least 18 years; comparing between PRP and other treatment or placebo or before–after receiving PRP; and had any of following outcomes including pain or functional score. Duplicate studies and studies with inadequate details for data extraction after failed contacting the corresponding authors for three times were excluded.

Eligible criteria for individual studies other than meta-analysis after the index date were RCTs, non-RCTs, cohort study, before–after study, or case series; recruiting ankle OA in adults aged at least 18 years; reporting PRP and other treatment or placebo or before–after receiving PRP; and had at least one outcome of interest (pain or functional score). Duplicate studies and studies with insufficient or inaccessible data after contacting the corresponding author for three attempts were excluded.

### Search strategy

PubMed and Scopus databases were searched from their inceptions to 31 January, 2023. The Preferred Reporting Items for Systematic review and Meta-Analysis (PRISMA) guideline was applied [14], and a review protocol was registered at PROSPERO (CRD42022297503). The clinical question was determined based on Population/patients, Intervention, Comparison, and Outcome (PICO) as follows: patients: “ankle osteoarthritis” and adult; interventions: PRP; comparisons: other intra-articular injection, conservative and surgical treatments; outcomes: pain and function. Search terms were set according to Patients, Interventions, and type of Studies (PIS), i.e. meta-analysis, and Patients, Interventions (PI) for individual studies (RCT, cohort, case series, before and after an index date of the latest published meta-analysis) with and without medical subject headings (MeSH) was applied where appropriate for PubMed. Search strategies were constructed by combining search terms of all domains (Table 1).

**Table 1:** Search strategies and search results

PIS	Order	Search terms	No. of publications	
			PubMed	Scopus
Patient (P)	#1	Search "ankle osteoarthritis"	654	838
	#2	Search "ankle arthrosis"	76	117
	#3	Search "ankle OA"	155	160
	#4	Search #1 OR #2 OR #3	765	980
Intervention (I)	#5	Search platelet-rich plasma	15,285	16,346
	#6	Search COBE Spectra OR Arthrex ACP	2417	4758
		OR PRGF-endoret OR ISTO Biologics,		
		OR Nuo Therapeutics OR		
		KYOCERA OR Medical PRP kit		
		OR JP200 OR SmartPrep OR		
		Plateltex OR OrthoGen OR Magellan		
	#7	Search #5 OR #6	17,554	19,484
PI	#8	Search #4 AND #7	25	56
Study design (S)	#9	Search meta-analysis	267,404	4,025,920
PIS	#10	Search #8 AND #9	2	33
PI latest*	#11	Search #4 AND #7	12	24

*Searching after an index date (February 2021) of the last update meta-analysis

### Selection and data collection

Individual studies included in previous meta-analyses and individual studies that were additionally identified from the last recent index searching date were selected for eligibility by two reviewers (SL, SP). Titles and abstracts were screened, and then, full articles were retrieved. The eligibility studies were reviewed and selected according to inclusion and exclusion criteria. Reference lists of the relevant articles were also checked. Disagreement between the two reviewers was resolved by consensus with the third author.

Two authors (SL, SP) independently extracted data using standardised data extraction forms. The forms comprised of study design, year of publication, number of patients, mean age, cause of ankle OA (primary, secondary), severe ankle OA (Kellgren–Lawrence classification [15]; KL 3–4 or Takakura classification 3–4 [16]), interventions, number of patients in each intervention, PRP techniques (preparation, dosage, administration), mean duration of follow-up, and outcomes. Authors were contacted if there were any insufficient data. Any disagreement was resolved by the third author.

### Data items

The outcomes of interest were pain measured by visual analog scale (VAS), functional scores by the American Orthopedic Foot and Ankle Society Score (AOFAS) [17], Ankle Osteoarthritis Scale (AOS) [17], or other foot and ankle-specific functional score, and ankle alignment. The secondary outcomes were quality of life and satisfaction score. VAS ranged 0–100 or 0–10 as based on the original articles [18], and the higher score indicates the more severe pain. The AOFAS score (ranges from worst to best of 0–100) consists of pain (one question, score 0–40), function (seven questions, score 0–50), and alignment (one question, score 0–10) [17]. The AOS measures pain (9 questions) and disability (9 questions) subscales and calculates into percentage ranged from 0 to 100 [17]. The foot and AOS ranges from 0 to 100; higher score reflects better with pain, symptoms, quality of life, activity of daily living, and sports and recreation subscales [18]. The Japanese Society for Surgery of the Foot ankle/hindfoot scale (JSSF) comprises pain (40 points), function (50 points), and alignment (10 points) [19]. For the quality of life, there were the 36-Item Short-Form Health Survey (SF-36), the total score from 0 (worst) to 100 (better health) [17], and the EuroQol 5-Dimension tool (EQ-5D) including mobility, self-care, usual activities, pain/discomfort, and anxiety/depression domains [18].

All outcomes were evaluated at 12 weeks with regard to the maximum treatment effect. Only minimally clinical important difference of AOS was available for ankle OA as 28/100 [17]. Possible adverse events such as pain at injection site; skin reactions; or soreness were also collected.

Intra-articular PRP injection was the intervention of interest. Possible comparators were intra-articular injection of inactive interventions (e.g. placebo, saline, sugar (prolotherapy)), conservative treatment (e.g. rest, shoe modification, exercise, physiotherapy), active interventions (e.g. botulinum toxin, hyaluronic acid, mesenchymal stem cell, corticosteroid or methylprednisolone), and surgery (e.g. arthroscopy, microfracture). Since PRP had various techniques, types of preparation, dosage, methods, and times of administration were taken into account. If there were not enough studies, the interventions were grouped as active and inactive comparators, in which active intervention was a combined PRP with mesenchymal stem cell (orthobiologic serum). Commercial brand lists of PRP [20] including previously used for tendinopathies [21] were also considered. Moreover, the standard guideline for PRP preparation from The Minimum Information for Studies evaluating Biologic in Orthopaedics (MIBO) [22] was assessed for each study. The 23-statement checklist encompasses the following domains: study design, patients’ details, intervention, whole blood processing and characteristics, PRP processing and characteristics, activation, delivery, postoperative care, and outcomes.

### Assessment of the risk of bias

The quality of the study was independently appraised by two authors using the Cochrane Collaboration’s tool for assessing risk of bias for randomised controlled trials (RoB 2) [23] for RCTs and risk of bias in non-randomised studies of interventions (ROBINS-I) [24] for non-RCTs. Any disagreement was discussed and resolved by the third evaluator.

### Assessment of the quality of recommendations

The Grading of Recommendations Assessment, Development and Evaluation (GRADE) [25] was used to determine the quality of evidences, i.e. study designs (RCT or observational studies), study limitations, inconsistent results, indirectness of evidence, imprecision, publication bias, large magnitude effects, plausible confounders reducing the treatment effect, dose–response relationship, and rating of the quality of evidences for each outcome. The strength of recommendation was expressed as for/against and strong/weak recommendation for the PRP treatment. The assessment was independently addressed by two authors. Any disagreement was discussed by consensus and resolved by the third evaluator.

### Data synthesis

A direct meta-analysis was performed comparing pain and functional scores between before and after receiving PRP. Heterogeneity was evaluated using Cochrane Q test and the I^2^-statistic. A standardised (SMD) or unstandardised mean difference (USMD) and 95% confidence interval (CI) were estimated and pooled across studies using a random-effects model if heterogeneity was present (*P*-value of *Q* test < 0.1 or *I*^2^ > 25%); otherwise, a fixed-effects model was applied. A meta-regression was applied to assess a source of heterogeneity by regressing the mean difference on platelet concentration and PRP volume. Sensitivity analysis was performed by excluding studies that might be a source of heterogeneity. Publication bias was assessed using Egger’s test [26] and a funnel plot [27]. In the case of significant Egger’s test or asymmetrical funnel plots, a contour-enhanced funnel plot was generated in order to differentiate publication biases from other causes [28].

A two-stage network meta-analysis was applied if the number of studies was sufficient to pool effects of PRP relative to active and non-active comparators as follows: First, a relative intervention effect, i.e. SMD/USMD, and its variance–covariance were estimated for each study; second, these effect sizes were pooled across studies using a multivariate meta-analysis. All possible relative treatment effects were estimated. Probability of being the best intervention was approximated using surface under the cumulative ranking curves (SUCRA). The consistency assumption was assessed using a design-by-treatment interaction model. A publication bias was evaluated using a comparison-adjusted funnel plot. A cluster rank was constructed for simultaneously considering probability of pain or functional scores, and possible adverse events such as pain, allergic reaction, skin discolouration, blood clot, and infection.

All analyses were performed using STATA Program 17.0. *P*-value < 0.05 was considered as non-statistical significance.

## Results

### Search results

**Fig. 1:**
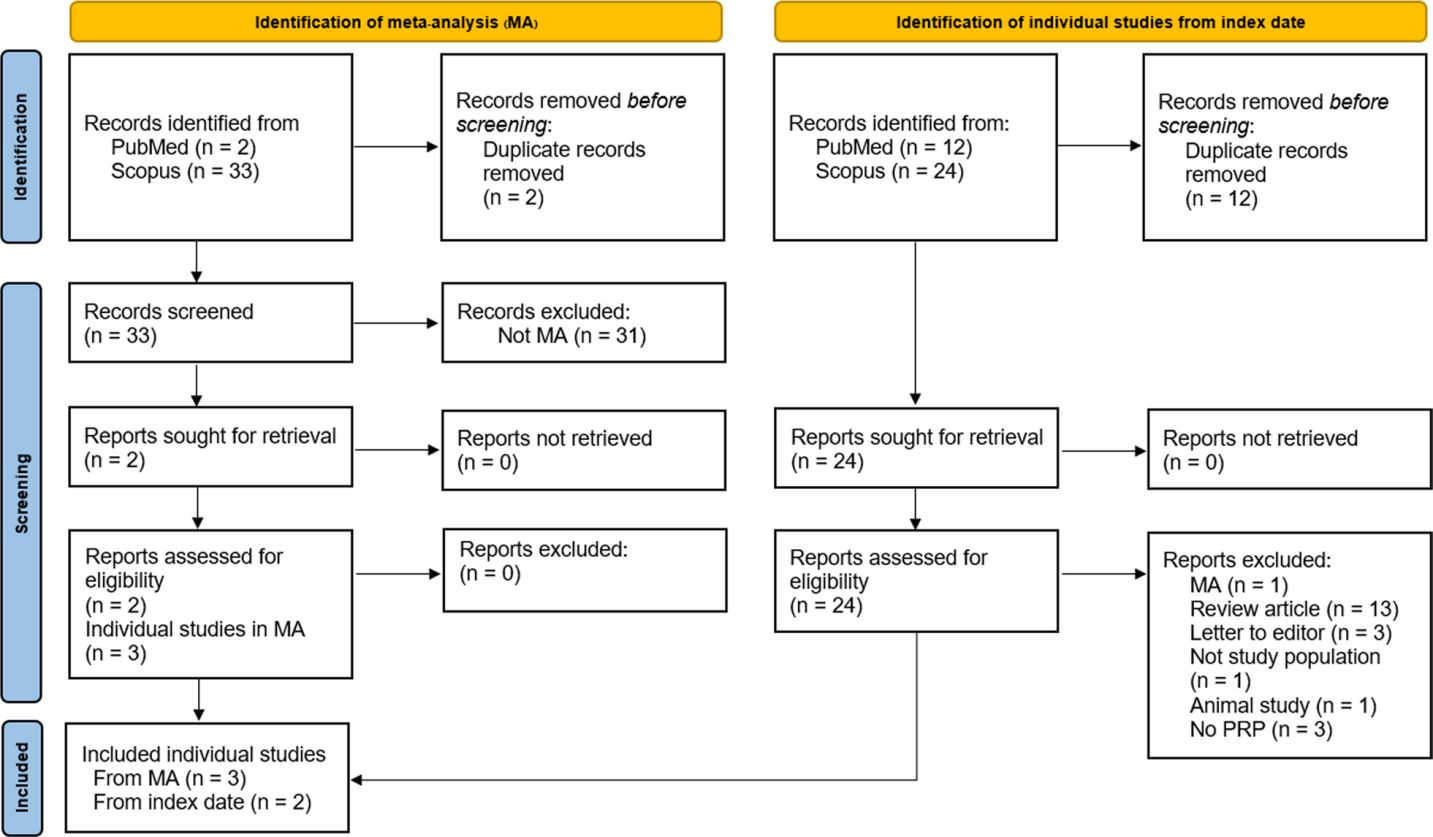
PRISMA flow diagram, *PRP* platelet-rich plasma

After considering patients and intervention terms, 81 studies were identified from PubMed and Scopus databases (Table 1). Then, type of studies “meta-analysis” was combined to search terms leaving 35 studies: two studies from PubMed and 33 studies from Scopus. Two duplicates and 31 studies other than meta-analysis were excluded. From two eligible meta-analyses [2, 4], three individual studies [12, 29, 30] met the inclusion criteria. There were 25 studies found from PubMed and 56 studies from Scopus before the index date. Three studies [12, 29, 30], the same as those from meta-analyses, were eligible. Additional 12 studies from PubMed and 24 studies from Scopus were identified from the index date. Twelve duplicates were removed, and 24 studies were screened. Twenty-two studies were excluded due to study designs, population, and intervention leaving two eligible studies [11, 13]. As a result, a total of five studies [11–13, 29, 30] were included in analysis (Fig. 1).

### Assessment of the risk of bias

The quality assessment demonstrated low risk of bias for the included randomised controlled trial [11] and serious to critical risk of bias among before–after studies [12, 13, 29, 30], Table 2.

**Table 2:** Quality assessment of included studies

Author	Domains					Overall
RoB-2	Randomisation process	Deviations from intended intervention	Missing outcome data	Measurement of the outcome	Selection of the reported results	
Paget [11]	Low	Low	Low	Low	Low	Low

ROBINS-I	Confounding	Selection of participants	Classification of intervention	Deviations from intended interventions	Missing data	Measurement of the outcome	Selection of the reported results	Overall
Angthong [30]	Serious	Critical	Serious	Moderate	Moderate	Serious	Moderate	Serious
Fukawa [12]	Serious	Serious	Low	Moderate	Low	Serious	Moderate	Serious
Repetto [29]	Critical	Moderate	Moderate	Moderate	Moderate	Serious	Moderate	Critical
Sun [13]	Serious	Low	Low	Moderate	Low	Serious	Moderate	Serious

### Assessment of the quality of recommendations

The GRADE quality of evidence for pain and functional outcomes is presented in Table 3. Quality of life had inadequate data to pool in the analysis. Overall quality was very low due to serious study limitations, unexplained heterogeneity, indirectness of evidence, and publication bias. The strength of recommendations for PRP treatment in ankle OA was weak.

**Table 3:** GRADE evidence for systematic review and meta-analysis of PRP treatment for ankle OA

Number of studies (participants)	Study limitations	Inconsistent results	Indirectness of evidence	Imprecision	Publication bias	Large magnitude effects	Plausible confounders	Dose–response relationship	Quality of evidence
*VAS (0–10) at 12 weeks*									
4 (127) 1 RCT 3 before–after	Serious limitations (3 studies)	Unexplained heterogeneity	Indirect	No important imprecision	Likely	USMD − 2.80 (95% CI: − 3.91, − 1.68)	Likely	Unlikely	+ , very low
*Function score (0–100) at 12 weeks*									
4 (127) 1 RCT 3 before–after	Serious limitations (3 studies)	No important inconsistency	Indirect	No important imprecision	Unlikely	Unlikely, SMD 1.73 (95% CI: 1.37, 2.09)	Likely	Unlikely	+ , very low
*Quality of life at 12 weeks*									
–	–	–	–	–	–	–	–	–	–

*GRADE* The Grading of Recommendations Assessment, Development and Evaluation, *PRP* platelet-rich plasma, *OA* osteoarthritis, *VAS* visual analog scale, *USMD* unstandardised mean difference, *CI* confidence interval, *SMD* standardised mean difference

### Study characteristics and results of individual studies

Characteristics of five included studies (total 184 patients for ankle OA, 132 patients for PRP, and 52 patients for placebo) are shown in Table 4. Of 5 studies published in 2013–2021, 1 RCT [11] compared PRP versus placebo, and 4 compared outcomes between before and after receiving PRP [12, 13, 29, 30]. The average age of participants was 50.8–59.3 years, and 25–60% of PRP injected cases were male. The number of primary ankle OA ranged from 0 to 100%. Severity according to KL 3–4 or Takakura 3–4 varied from 10 to 100%. Average follow-up period was 24 to 212 weeks. Most outcome measures were VAS, and AOFAS for the outcomes. Details of PRP preparation, dosage, frequency, and administration are presented in Table 5. All included studies [11–13, 29, 30] reported 14–19 out of 23 MIBO checklists mostly on details of recipient, injury, whole blood processing, PRP processing and format, delivery, post-operative care, and outcomes (Additional file 1: Table S1).

**Table 4:** Baseline characteristics of included studies

Author	Country	Year	Design	Mean age (y)	OA, primary (N)	Severe OA Takakura/ KL 3–4 (%)	PRP (N)	Comparator (N)	Male (%)	FU Time (week)	Outcome
Angthong [30]	Thailand	2013	Retrospective Before–after descriptive	50.8	5, NA	20	5	No	60	192	VAS-FA SF-36
Fukawa [12]	Japan	2017	Prospective Before–after descriptive	59.3	20, 20	10	20	No	25	24	VAS JSSF SAFE-Q
Repetto [29]	Italy	2017	Retrospective Before–after descriptive	57.5	20, 0	100	20	No	60	212	VAS FADI
Paget [11]	Netherlands	2021	RCT	55.6	100, 1	100	48	Placebo (52)	54	26	VAS AOFAS FAO AOS AAS SF-36 EQ5D3L
Sun [13]	Taiwan	2021	Prospective Before–after descriptive	55.5	39, NA	28.2	39	No	43	24	VAS AOFAS AOS

*OA* osteoarthritis, *NA* not available, *KL* Kellgren–Lawrence, *PRP* platelet-rich plasma, *FU* follow up, *RCT* randomised controlled trial, *VAS-FA* visual analog scale foot and ankle, *SF-36* the 36-Item Short Form Health Survey, *VAS* visual analog scale, *JSSF* the Japanese Society for Surgery of the Foot ankle/hindfoot scale, *SAFE-Q* the Self-Administered Foot Evaluation Questionnaire, *FADI* the Foot and Ankle Disability Index, *AOFAS* the American Orthopaedic Foot and Ankle Society score, *FAO* Foot and Ankle Outcome Score, *AOS* Ankle Osteoarthritis Score, *AAS* Ankle Activity Score (AAS), *EQ5D3L* The 3-Level EuroQol 5-Dimension (EQ-5D-3L)

**Table 5:** Platelet-rich plasma preparation and administration

PRP study	Blood (ml)	Equipment	Dosage (ml)	Frequency (interval)	Guidance	Anesthetics	Ankle injection
Angthong [30]	9–10	Arthrex ACP	3	1	Flu/US	1% lidocaine	Peri-lesion
Fukawa [12]	200	Blood-Separator	2	3 (2 weeks)	US	–	Anteromedial
Repetto [29]	450	Hettich Zentrifugen	3	4 (1 week)	No	No	Anteromedial
Paget [11]	15	Arthrex	2	2 (6 weeks)	US	No	Anteromedial & anterolateral
Sun [13]	7	PLTenus PLUS	3	1	No	No	Intra-articular

*PRP* platelet-rich plasma, *Flu* fluoroscope, *US* ultrasound

### Synthesis of results

#### Pain score

**Fig. 2:**
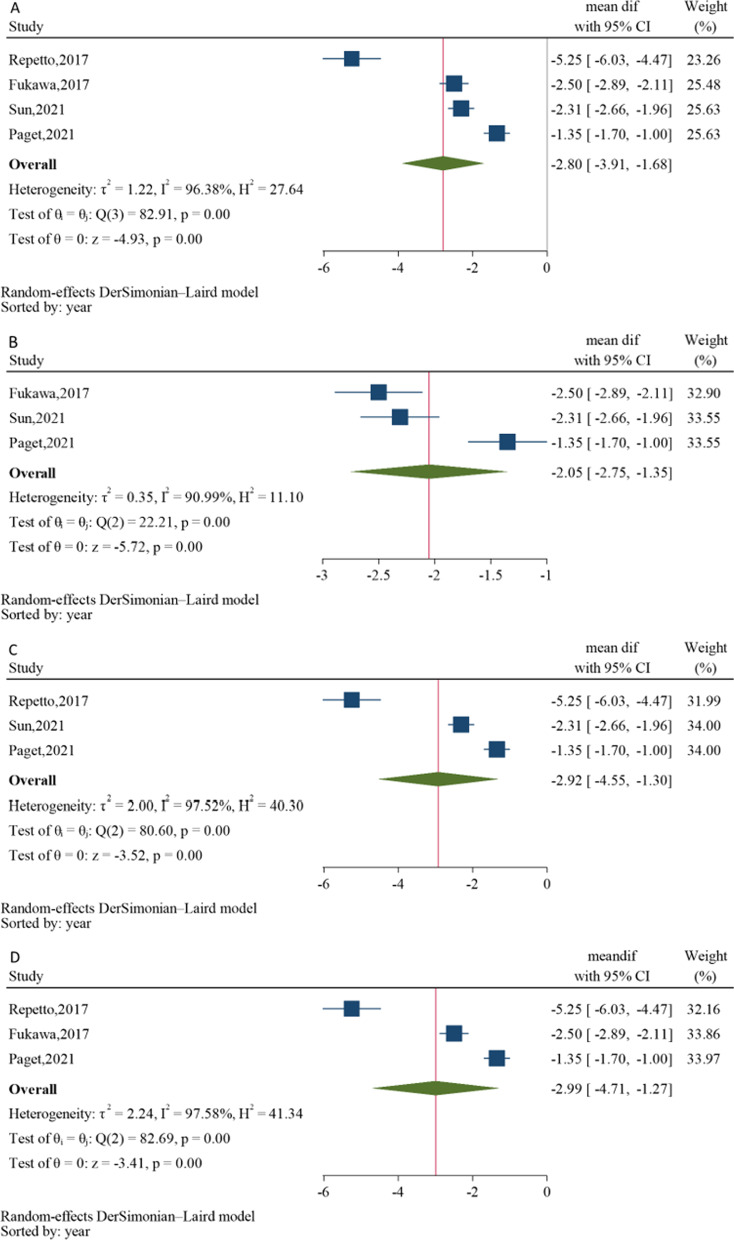
Forest plots of visual analog scale at 12 weeks comparing between before and after receiving platelet-rich plasma (PRP) for **A** overall pooling and sensitivity analysis by excluding, **B** high-dose PRP, **C** primary OA, and **D** severe OA < 50%

Regarding the hypothesis that PRP administration would reduce pain for ankle OA, the difference of VAS between before and after PRP injection was estimated. One study reported different outcome measurement as VAS-FA [30], leaving four studies [11–13, 29] for the meta-analysis. Data pooling across 4 studies [11–13, 29] indicated significant VAS reduction at 12 weeks after receiving PRP (the pooled USMD of − 2.80, 95% CI − 3.91, − 1.68; *P* < 0.001), Fig. 2A. However, treatment effects were highly varied across studies (*Q* = 82.91; *P* < 0.001, *I*^2^ 96.38%). A meta-regression was applied to assess if platelet concentration and PRP volume contributed to VAS score changes. Only PRP volume, but not platelet concentration, was significantly associated with VAS mean difference. Every increment in PRC volume by one unit would significantly reduce VAS score by 0.39 (95% CI − 0.61, − 0.16), Additional file 1: Fig. S1. However, including platelet concentration and PRP volume could not reduce the degree of heterogeneity with the *I*^2^ of 97.45% and 93.01%, respectively (Additional file 1: Table S2).

A sensitivity analysis was performed by excluding studies possibly accounting for heterogeneity, i.e. high dosage of PRP [29], primary ankle OAs [12], or low number (< 50%) of severe ankle OA (KL 3–4 or Takakura 3–4) [13], but none of them could reduce the heterogeneity, Fig. 2B–D. After excluding high-dosage study, 1–3 doses of PRP minimised VAS more than before treatment (the pooled USMD of − 2.05, 95%CI − 2.75, − 1.35, *P* < 0.001) with *I*^2^ 90.99% (Fig. 2B). Considering only traumatic OA, VAS score was declined after receiving PRP (the pooled USMD of − 2.92, 95%CI − 4.55, − 1.30) and *I*^2^ 97.52%, Fig. 2C. The studies reported ≥ 50% of severe ankle OA showed significant pain relief after PRP treatment (the pooled USMD of − 2.99, 95%CI − 4.71, − 12.7) with *I*^2^ = 97.58%, Fig. 2D.

In addition, a subgroup analysis by age < 56 years and ≥ 56 years resulted in USMD of − 3.85 (95%CI − 6.55, − 1,66) and − 1.83 (95% CI − 2.77, − 0.89), Additional file 1: Fig. S2.

The publication bias was assessed. The funnel plot (Additional file 1: Fig. S3A) and Egger’s test (coefficient = − 15.18, *P* < 0.001) showed asymmetry. Consequently, the contour-enhanced funnel plot was constructed and suggested that asymmetry might be caused by heterogeneity and publication bias (Additional file 1: Fig. S3B).

#### Functional score

**Fig. 3:**
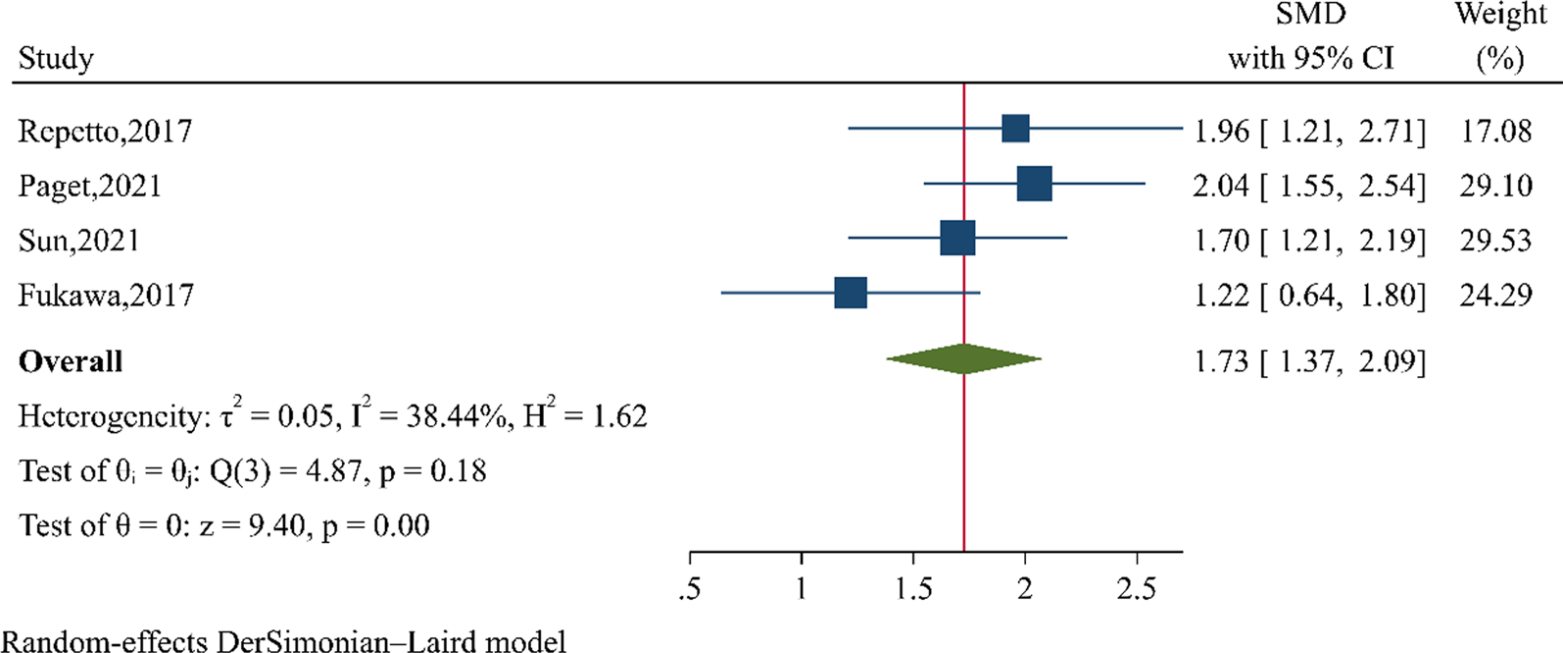
Forest plot of functional score at 12 weeks pooling standardised mean difference between before and after platelet-rich plasma (PRP) injection

**Fig. 4:**
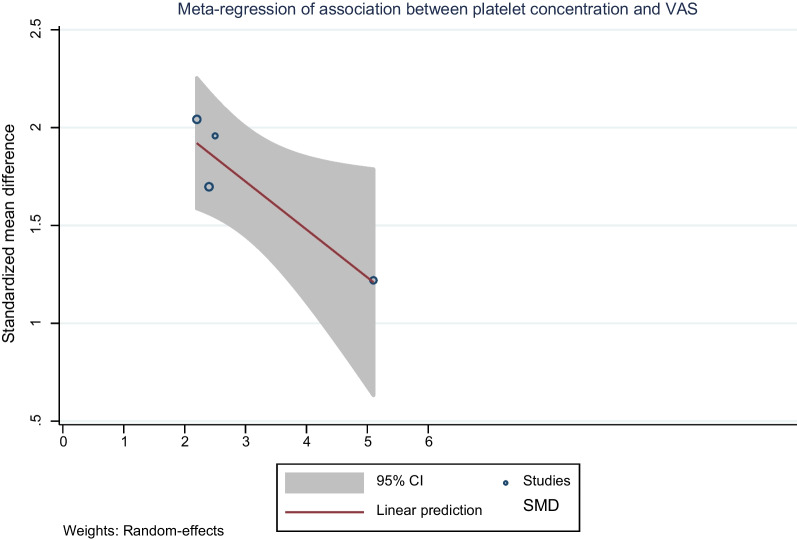
Meta-regression of the association between platelet concentration and functional score

The functional scores were reported in 4 studies [11–13, 29] using AOFAS [11, 13], Foot and Ankle Disability Index (FADI) [29], and JFFS [12]. The hypothesis that PRP administration would improve function in ankle OA was tested by including these 4 studies [11–13, 29] into the meta-analysis. SMD was estimated and pooled across 4 studies [11–13, 29] indicating PRP significantly improved functional score (the pooled SMD of 1.73, 95% CI 1.37, 2.09; *P* < 0.001) with *Q* = 4.87, *P* = 0.18; *I*^2^ = 38.44% when compared with before treatment (Fig. 3). A meta-regression was applied for 4 included studies [11–13, 29] and suggested that platelet concentration had significant negative association with functional score. One unit increasing in platelet concentration would decrease function by 0.24, Fig. 4. Neither Egger’s test (coefficient = 0.19, *P* = 0.966) nor funnel plot showed asymmetry for functional score (Additional file 1: Fig. S4).

### Quality of life

Three studies assessed reported quality of life using SF-36 [11, 29, 30], the Self-Administered Foot Evaluation Questionnaire (SAFE-Q) [12], and The 3-Level EQ-5D (EQ-5D-3L) [11]. Meta-analysis was unable to be performed due to inadequate number of studies for data pooling.

Adverse events among PRP were identified in three studies [11–13] as knee pain (4%) [11], lower limb muscle soreness (26%) [11], pain at injection site (12.8%) [13], and pain with swelling at injection site (5%) [12]. Lower limb muscle soreness was found in about 5% of placebo injections [11]. No serious adverse events were documented in both PRP and placebo groups.

## Discussion

PRP has not been specifically reviewed for ankle OA. Various PRP preparations were used, i.e. commercial/local equipment, 2–3 ml, 1–4 doses, 0–6 weeks of interval, with or without ultrasound/fluoroscopic guidance, local anesthesia, or peri-lesion/ankle joint injection. According to the hypothesis that PRP administration would improve pain and function among ankle OA patients, the results showed significant VAS reduction and functional score improvement after receiving treatment for 12 weeks. However, pooling PRP effects were based on high heterogeneity that could not be minimised by sensitivity and subgroup analyses by age, dosage, primary ankle OA, or severity.

Two previous meta-analyses [4, 5] of ankle OA have specifically focused on hyaluronic acid versus saline injection [4], placebo, botulinum toxin A, or progressive ankle exercise [5]. This systematic review and meta-analysis are the first study which has summarised PRP effect on ankle OA. PRP could not only be benefit in reducing VAS, but was also advantageous in functional score improvement by 1.73 SMD (Fig. 3) which was approximately 10–12 scores of AOFAS [11, 13], 20 scores of FADI [29], and 13 scores of JSSF [12] (Fig. 6). These differences reached minimal clinical significance at 12 weeks of follow-up. The short-term effects may help patients return to their activities with possibly better quality of life. However, the included well-designed RCT [11] did not find significant difference of either VAS at 6, 12, and 26 weeks or AOFAS, SF-36 at 6 and 26 weeks between two 2-ml PRP and placebo. Placebo increased by as many as 11 scores of AOFAS by 26 weeks after injection.

### PRP preparation

PRP preparation forms might also effect on the treatment outcomes. Meta-regression of 4 included studies [11–13, 29] showed platelet concentration was a source of heterogeneity for VAS with *I*^2^ 97.45% (Additional file 1: Table S2). The PRP is taken from centrifugation of the individual’s whole blood, which is different in terms of blood component, in both number of cells and growth factors. High platelet counts (600,000 cells/mm^3^) and low leucocytes (< 1000 cells/mm^3^) after filtration are mandatory for the optimal outcomes [21, 29].

The MIBO emphasised reporting cellular components from whole blood and deliverable process of PRP to understand influential characteristics of individual preparation [22]. Even though all included studies detailed deliverable processes, only three studies [12, 13, 29] reported the amounts of platelet counts (Additional file 1: Table S1). Their platelet concentration of PRP was moderate at 2 to 5 times of the blood platelet count, i.e. range 250,000–900,000 cells/mm^3^ [29], and 1,310,400 ± 667,0000 cells/mm^3^ [12]. The other two studies [11, 30] used standard commercial administration and did not elaborate the composition of PRP. However, a previous scoping review summarised commercial PRP with platelet counts of 2,310,000 ± 524,000 cells/mm^3^ and leucocyte counts of 10,000 ± 4.500 cell/mm^3^ [31].

The number of studies of PRP treatment in OA ankle has been scarce. In this review, three before–after series supported that use of PRP was safe and effective [12, 13, 29], while another small study suggested the PRP injection as optional [30]. Conversely, the included RCT [11] showed no benefits of pain relief and functional improvement compared to the placebo. In order to make therapeutic recommendations of PRP for ankle OA, further well-designed RCTs should be conducted in accordance with the MIBO guideline.

### Injury details

This sensitivity analysis did not find the relationship between traumatic OA or severe ankle OA and VAS. The explanation might be a limitation of previous surgical information, and reliability of Takakura and Kellgren classification [32]. Most ankle OA is secondary to fractures and ligament injuries [33], and usually associates with coronal and sagittal planes’ deformities. Abnormal biomechanics might affect the results and should be addressed before PRP application. Though existing literatures showed benefits of corrective osteotomy associated with ankle OA [34], the additive effect of PRP injection following these joint preserving surgeries has not been investigated.

Numerous classifications of ankle OA are not designed specifically to associate with the primary or secondary causes. Claessen et al. [32] reported fair interobserver agreement in van Dijk classification and documented poor agreement in Takakura and Kellgren classification. The standardised classification to clarify the severity of the diseases must include abnormal biomechanics, particularly in post-traumatic ankle OA.

### Whole blood processing and characteristics

Whole blood platelets, differential leukocytes, and red blood cells should be analysed in all samples. Unfortunately, none of the included studies reported whole blood characteristics. Type and dose of anticoagulant is another concern. By chelating Ca+, ethylenediaminetetraacetic acid (EDTA) might disrupt function of platelets and might reduce the growth factors in the PRP prepared from EDTA-anticoagulated whole blood [35]. Thus, the adverse effect of additives must be considered in the process of whole blood taking.

### PRP processing and characteristics

Three studies [11, 12, 29] from this review quantified PRP characteristics and two of them [12, 29] reported storage temperature at -30 and -80 degrees Celsius, respectively (Additional file 1: Table S1). As the preparation technique and components of PRP seem to be the most important factors affecting the outcomes of treatment of musculoskeletal disorders with PRP injection, there is no universally accepted classification of PRP available. Many classifications have been proposed which mostly involved the concentration of platelets, presence of red and white blood cells, and use of exogenous activators before injection [36]. There are available commercial kits used to prepare the PRP which produce variety of the final preparation in terms of platelets’ concentration [21]. Different kits need different volumes of whole blood and require particular centrifugation techniques. Quantitative reporting on all main components of PRP should be determined. Moreover, the number of platelets, white blood cells, and percentage of neutrophils have to be reported.

Two included studies [12, 29] described only storage temperature without PRP processing conditions. Temperature during PRP preparation might provide the positive effect to the PRP. Du et al. [37] presented PRP processing under low temperature (4 degrees Celsius) with a double spinning technique compared to a contemporary method. They reported higher concentration of platelets. More platelets and growth factors were trapped in the natural fibre scaffolding after activation by rewarming PRP preparation from lower temperature.

### Activation

After PRP injection into the tissue, the natural process of activation is commenced forming the fibrin network, and the plasma becomes the gel-like fibrin clot [38]. Some injection techniques encouraged platelets’ activation, with thrombin or calcium chloride, before delivering to the tissue. The time between activation and injection is very critical because most of the growth factors are secreted within 10 min after activation and almost 100% of them are secreted within an hour [36]. Three studies from this review stated the activation process. Fukawa et al. [12] added calcium chloride hydrate just before PRP injection. Sun et al.,[13] used citrate without providing time to deliver. Both studies reported significant pain reduction after injection. Paget et al. [11] did not use thrombin, calcium, or citrate substances. The authors found non-significant different pain between PRP and placebo.

### Delivery and outcomes

Total volume of injection has been recognised as one of influencing factors affecting the treatment outcome. Regarding to meta-regression of four included studies [11–13, 29], PRP volume was found as sources of heterogeneity for VAS and functional score with *I*^2^ 93.01% and 58.93%, respectively. In the study by Repetto et al. [29], the authors provided four injections of PRP (total 12 ml), while Fukawa et al. [12] administered three injections (total 6 ml). Both studies reported positive effects on pain and functional outcomes. However, the optimal doses and injection interval in particular ankle OA have not been investigated.

Age, immunity, metabolic disorders, and concomitant medication possibly impacted to cellular impairment, collagen deformation, microbiota changes, cellular viability and may contribute to therapeutic outcomes of PRP [39]. From four included studies [11–13, 29], average age ranged from 50.8 to 59.3 years, no immune or metabolic abnormalities were reported, and no local anesthesia/cytotoxic agents were used. Another study [30] excluded patients with systemic diseases, but lidocaine was applied prior to PRP injection.

Future studies investigating the additive effects of collective doses are needed to support multiple injections. Intrinsic and extrinsic factors affecting PRP quality have to be explored. Moreover, standardised clinical and imaging outcomes should be assessed and compared between studies.

### Strengths and limitations

Since this review was based on secondary data, the quality of evidence and data retrieval might be limited and affect the clinical application. Data were gathered as much as possible, and were all researchers agreed in searching, selection, and extraction by consensus. The quality of the studies, publication biases, and heterogeneity were assessed with standard statistical analysis to achieve the best available evidence. Limitations of this study are including descriptive case series with serious to critical risk of biases, publication biases or heterogeneity of the VAS outcome, very low-GRADE quality of evidences, inadequate data of quality of life for analysis and focused only on the maximum treatment effects at 12 weeks of follow-up. Included PRP studies in this review seemed to deliver a similar effect size as that of placebo from the previous RCT [11]. Regarding the risk of biases towards positive results, pain and functional improvement from these PRP preparations might not be superior to placebo. The functional outcomes were varied across the studies. However, there was no publication bias, and the standardised mean difference was applied for appropriate comparisons.

### Clinical application

Treatments of ankle OA with various PRP preparations seemed to be effective, in terms of pain and function, when compared with before treatment. With very low quality of evidences, high costs, and diverging settings, PRP is weakly recommended for ankle OA as an alternative or adjunct therapy after failed conservative treatment. Its benefits would be attained approximately 12 weeks after injection with acceptable minor complications.

## Conclusion

With regard to the hypothesis, the best available evidence of pooling different PRPs for ankle OA demonstrated only short-term, before–after pain, and functional improvement. Its benefit was similar to placebo effects of the included RCT. Further modified PRP preparation or limited use as alternative therapy may apply for ankle OA. The future studies investigating the outcomes of PRP injection for ankle OA should follow the MIBO consensus to make the processing of PRP preparation reproducible and to make the comparison of the outcomes between studies possible.

## Supplementary Information

**Additional file 1**: **Table S1** Minimum Reporting Requirements for Clinical Studies Evaluating PRP Checklists for included studies. **Table S2** Meta-regression of factors related to pain and functional score. **Fig. S1** Meta-regression of association between platelet-rich plasma (PRP) volume and visual analog scale (VAS).  **Fig. S2** Forest plots of visual analog scale at 12 weeks: A subgroup analysis by age groups. **Fig. S3** A Funnel plot and B contour-enhanced funnel plot for visual analog scale. **Fig. S4** Funnel plot for functional scores, SMD = standardised mean difference.

## Data Availability

The review protocol, and datasets used and/or analysed during the current study are available from the corresponding author upon reasonable request.

## References

[1] Murray C, Marshall M, Rathod T, Bowen CJ, Menz HB, Roddy E (2018). Population prevalence and distribution of ankle pain and symptomatic radiographic ankle osteoarthritis in community dwelling older adults: a systematic review and cross-sectional study. PLoS ONE.

[2] Boffa A, Previtali D, Di Laura FG, Vannini F, Candrian C, Filardo G (2021). Evidence on ankle injections for osteochondral lesions and osteoarthritis: a systematic review and meta-analysis. Int Orthop.

[3] Chang KV, Hsiao MY, Chen WS, Wang TG, Chien KL (2013). Effectiveness of intra-articular hyaluronic acid for ankle osteoarthritis treatment: a systematic review and meta-analysis. Arch Phys Med Rehabil.

[4] Vannabouathong C, Del Fabbro G, Sales B, Smith C, Li CS, Yardley D (2018). Intra-articular injections in the treatment of symptoms from ankle arthritis: a systematic review. Foot Ankle Int.

[5] Witteveen AG, Hofstad CJ, Kerkhoffs GM (2015). Hyaluronic acid and other conservative treatment options for osteoarthritis of the ankle. Cochrane Database Syst Rev.

[6] Evans A, Ibrahim M, Pope R, Mwangi J, Botros M, Johnson SP (2020). Treating hand and foot osteoarthritis using a patient's own blood: a systematic review and meta-analysis of platelet-rich plasma. J Orthop.

[7] Guney A, Yurdakul E, Karaman I, Bilal O, Kafadar IH, Oner M (2016). Medium-term outcomes of mosaicplasty versus arthroscopic microfracture with or without platelet-rich plasma in the treatment of osteochondral lesions of the talus. Knee Surg Sports Traumatol Arthrosc.

[8] Görmeli G, Karakaplan M, Görmeli CA, Sarıkaya B, Elmalı N, Ersoy Y (2015). Clinical effects of platelet-rich plasma and hyaluronic acid as an additional therapy for talar osteochondral lesions treated with microfracture surgery: a prospective randomized clinical trial. Foot Ankle Int.

[9] Mei-Dan O, Carmont MR, Laver L, Mann G, Maffulli N, Nyska M (2012). Platelet-rich plasma or hyaluronate in the management of osteochondral lesions of the talus. Am J Sports Med.

[10] Malahias MA, Roumeliotis L, Nikolaou VS, Chronopoulos E, Sourlas I, Babis GC (2021). Platelet-rich plasma versus corticosteroid intra-articular injections for the treatment of trapeziometacarpal arthritis: a prospective randomized controlled clinical trial. Cartilage.

[11] Paget LDA, Reurink G, de Vos RJ, Weir A, Moen MH, Bierma-Zeinstra SMA (2021). Effect of platelet-rich plasma injections vs placebo on ankle symptoms and function in patients with ankle osteoarthritis: a randomized clinical trial. JAMA.

[12] Fukawa T, Yamaguchi S, Akatsu Y, Yamamoto Y, Akagi R, Sasho T (2017). Safety and efficacy of intra-articular injection of platelet-rich plasma in patients with ankle osteoarthritis. Foot Ankle Int.

[13] Sun SF, Hsu CW, Lin GC, Lin HS, Chou YJ, Wu SY (2021). Efficacy and safety of a single intra-articular injection of platelet-rich plasma on pain and physical function in patients with ankle osteoarthritis: a prospective study. J Foot Ankle Surg.

[14] Page MJ, McKenzie JE, Bossuyt PM, Boutron I, Hoffmann TC, Mulrow CD, et al. The prisma 2020 statement: an updated guideline for reporting systematic reviews. 2021;372:n71. 10.1136/bmj.n71 PMC8005924 33782057

[15] Kellgren JH, Lawrence JS (1957). Radiological assessment of osteo-arthrosis. Ann Rheum Dis.

[16] Takakura Y, Tanaka Y, Kumai T, Tamai S (1995). Low tibial osteotomy for osteoarthritis of the ankle. Results of a new operation in 18 patients. J Bone Joint Surg Br.

[17] Madeley NJ, Wing KJ, Topliss C, Penner MJ, Glazebrook MA, Younger AS (2012). Responsiveness and validity of the sf-36, ankle osteoarthritis scale, aofas ankle hindfoot score, and foot function index in end stage ankle arthritis. Foot Ankle Int.

[18] Shazadeh Safavi P, Janney C, Jupiter D, Kunzler D, Bui R, Panchbhavi VK (2019). A systematic review of the outcome evaluation tools for the foot and ankle. Foot Ankle Spec.

[19] Niki H, Aoki H, Inokuchi S, Ozeki S, Kinoshita M, Kura H (2005). Development and reliability of a standard rating system for outcome measurement of foot and ankle disorders II: interclinician and intraclinician reliability and validity of the newly established standard rating scales and Japanese Orthopaedic Association rating scale. J Orthop Sci.

[20] Andia I, Maffulli N (2018). A contemporary view of platelet-rich plasma therapies: moving toward refined clinical protocols and precise indications. Regen Med.

[21] Fitzpatrick J, Bulsara MK, McCrory PR, Richardson MD, Zheng MH (2017). Analysis of platelet-rich plasma extraction: variations in platelet and blood components between 4 common commercial kits. Orthop J Sports Med.

[22] Murray IR, Geeslin AG, Goudie EB, Petrigliano FA, LaPrade RF (2017). Minimum information for studies evaluating biologics in orthopaedics (mibo): platelet-rich plasma and mesenchymal stem cells. J Bone Joint Surg Am.

[23] Sterne JAC, Savović J, Page MJ, Elbers RG, Blencowe NS, Boutron I (2019). Rob 2: a revised tool for assessing risk of bias in randomised trials. BMJ.

[24] Sterne JA, Hernán MA, Reeves BC, Savović J, Berkman ND, Viswanathan M (2016). Robins-i: a tool for assessing risk of bias in non-randomised studies of interventions. BMJ.

[25] Guyatt GH, Oxman AD, Kunz R, Vist GE, Falck-Ytter Y, Schünemann HJ (2008). What is “quality of evidence” and why is it important to clinicians?. BMJ.

[26] Page MJ, Sterne JAC, Higgins JPT, Egger M (2021). Investigating and dealing with publication bias and other reporting biases in meta-analyses of health research: a review. Res Synth Methods.

[27] Sterne JA, Egger M (2001). Funnel plots for detecting bias in meta-analysis: guidelines on choice of axis. J Clin Epidemiol.

[28] Peters JL, Sutton AJ, Jones DR, Abrams KR, Rushton L (2008). Contour-enhanced meta-analysis funnel plots help distinguish publication bias from other causes of asymmetry. J Clin Epidemiol.

[29] Repetto I, Biti B, Cerruti P, Trentini R, Felli L (2017). Conservative treatment of ankle osteoarthritis: Can platelet-rich plasma effectively postpone surgery?. J Foot Ankle Surg.

[30] Angthong C, Khadsongkram A, Angthong W (2013). Outcomes and quality of life after platelet-rich plasma therapy in patients with recalcitrant hindfoot and ankle diseases: a preliminary report of 12 patients. J Foot Ankle Surg.

[31] Pachito DV, Bagattini ÂM, de Almeida AM, Mendrone-Júnior A, Riera R (2020). Technical procedures for preparation and administration of platelet-rich plasma and related products: a scoping review. Front Cell Dev Biol.

[32] Claessen FM, Meijer DT, van den Bekerom MP, Gevers Deynoot BD, Mallee WH, Doornberg JN (2016). Reliability of classification for post-traumatic ankle osteoarthritis. Knee Surg Sports Traumatol Arthrosc.

[33] Valderrabano V, Horisberger M, Russell I, Dougall H, Hintermann B (2009). Etiology of ankle osteoarthritis. Clin Orthop Relat Res.

[34] Barg A, Pagenstert GI, Horisberger M, Paul J, Gloyer M, Henninger HB (2013). Supramalleolar osteotomies for degenerative joint disease of the ankle joint: indication, technique and results. Int Orthop.

[35] Davlouros P, Xanthopoulou I, Mparampoutis N, Giannopoulos G, Deftereos S, Alexopoulos D (2016). Role of calcium in platelet activation: novel insights and pharmacological implications. Med Chem.

[36] Rossi LA, Murray IR, Chu CR, Muschler GF, Rodeo SA, Piuzzi NS (2019). Classification systems for platelet-rich plasma. Bone Joint J..

[37] Du L, Miao Y, Li X, Shi P, Hu Z (2018). A novel and convenient method for the preparation and activation of prp without any additives: temperature controlled prp. Biomed Res Int.

[38] Arnoczky SP, Sheibani-Rad S (2013). The basic science of platelet-rich plasma (prp): what clinicians need to know. Sports Med Arthrosc Rev.

[39] Andia I, Maffulli N (2018). Some patients (and some of us) respond better to some biological therapies: the as yet unsolved conundrum. J Orthop Traumatol.

