# Supplementary material for: Platelet-rich plasma injection for the treatment of ankle osteoarthritis: a systematic review and meta-analysis
Source: J Orthop Surg Res. 2023 May 19;18:373. doi: 10.1186/s13018-023-03828-z (PMC10197236; doi:10.1186/s13018-023-03828-z)
Supplement: Supplementary file 1 — Additional file 1: Table S1 Minimum Reporting Requirements for Clinical Studies Evaluating PRP Checklists for included studies. Table S2 Meta-regression of factors related to pain and functional score. Fig. S1 Meta-regression of association between platelet-rich plasma (PRP) volume and visual analog scale (VAS). Fig. S2 Forest plots of visual analog scale at 12 weeks: A subgroup analysis by age groups. Fig. S3 A Funnel plot and B contour-enhanced funnel plot for visual analog scale. Fig. S4 Funnel plot for functional scores, SMD = standardised mean difference. [file 13018_2023_3828_MOESM1_ESM.docx]

**Additional file 1**

**Table S1** Minimum Reporting Requirements for Clinical Studies Evaluating PRP checklists for included studies

| **Topic** | **Item/Authors,year** | Angthong, 2013 | Fukawa, 2017 | Repetto, 2017 | Paget, 2021 | Sun, 2021 |
| --- | --- | --- | --- | --- | --- | --- |
| Study design | Conduct with guidelines | - | - | - | + | - |
|  | Ethical approval | - | + | - | + | + |
| Recipient | Demographics | + | + | + | + | + |
|  | Comorbidity | + | + | + | + | + |
|  | Anti-inflammatory drug | + | + | + | + | + |
| Injury | Diagnosis | + | + | + | + | + |
|  | Preoperative imaging | + | + | + | + | + |
|  | Previous treatment | + | + | + | + | + |
| Intervention | Intervention description | + | - | + | + | + |
|  | Operative findings | - | - | - | - | - |
| Whole blood | Processing | + | + | + | + | + |
|  | Characteristics | - | - | - | - | - |
| PRP | Processing description | + | + | + | + | + |
|  | Platelet recovery rate | - | - | + | - | - |
|  | Storage temperature, light | - | + | + | - | - |
|  | Time processing, delivery | - | + | - | + | - |
| PRP | Format | + | + | + | + | + |
|  | Platelets, differential cells | - | + | + | + | - |
| Activation | Activation description | - | + | - | + | + |
| Delivery | Point of delivery | + | + | + | + | + |
|  | Delivery description | + | + | + | + | + |
| Postoperative care | Rehabilitation protocol | + | + | + | + | - |
| Outcomes | Assessment, functional outcomes, complications | + | + | + | + | + |

PRP = platelet-rich plasma, + = present, - = no report

**Table S2** Meta-regression of factors related to pain and functional score

| Outcome | Sources of heterogeneity | No. studies | I^2^ (%) |
| --- | --- | --- | --- |
| Pain score | Platelet concentration | 4 | 97.45 |
|  | PRP volume | 4 | 93.01 |
| Functional score | Platelet concentration | 4 | 0.00 |
|  | PRP volume | 4 | 58.93 |

PRP = platelet-rich plasma

**Fig. S1** Meta-regression of the association between platelet-rich plasma (PRP) volume and visual analog scale (VAS)

**
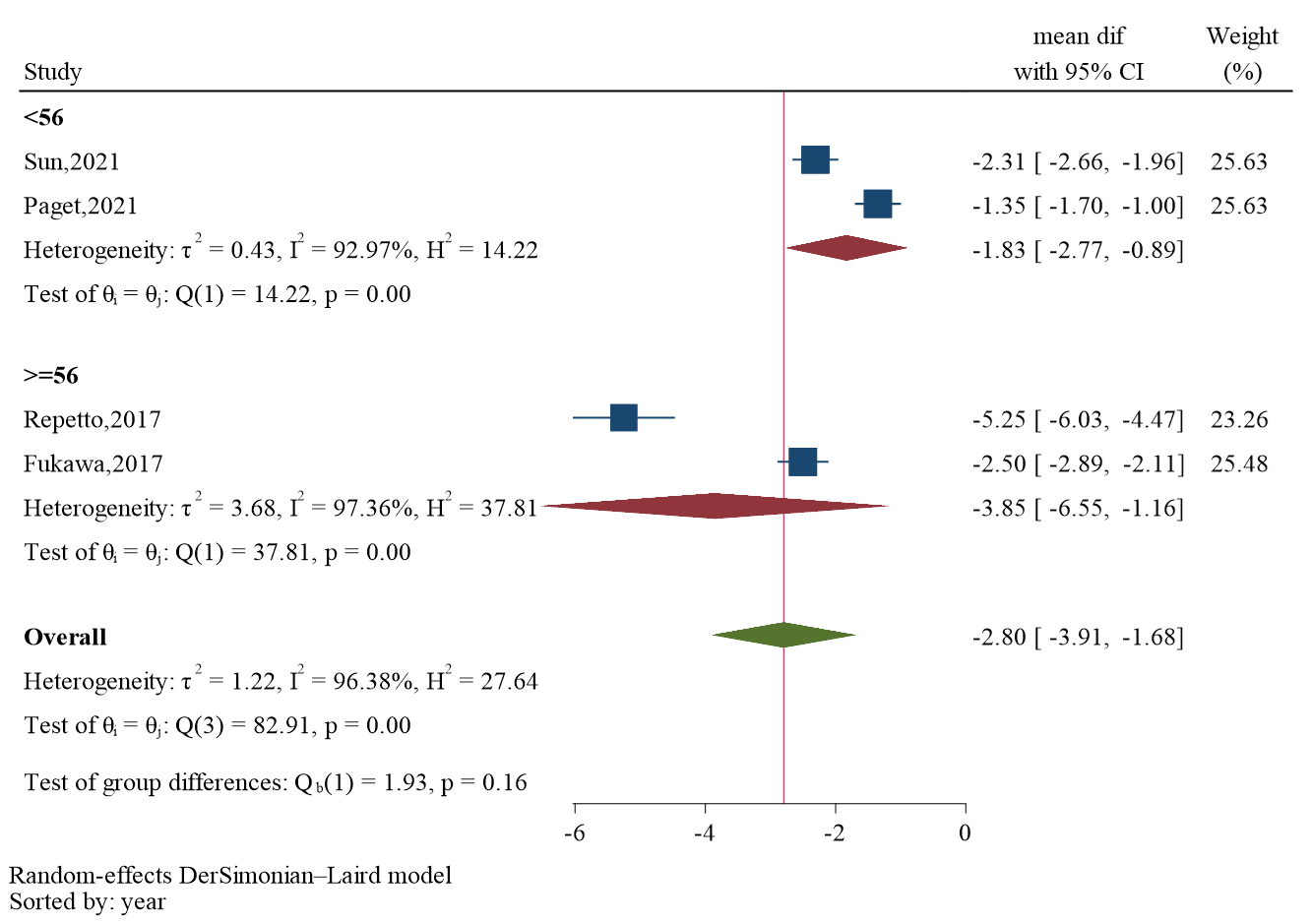
**

**Fig. S2** Forest plots of visual analog scale at 12 weeks: A subgroup analysis by age groups

**
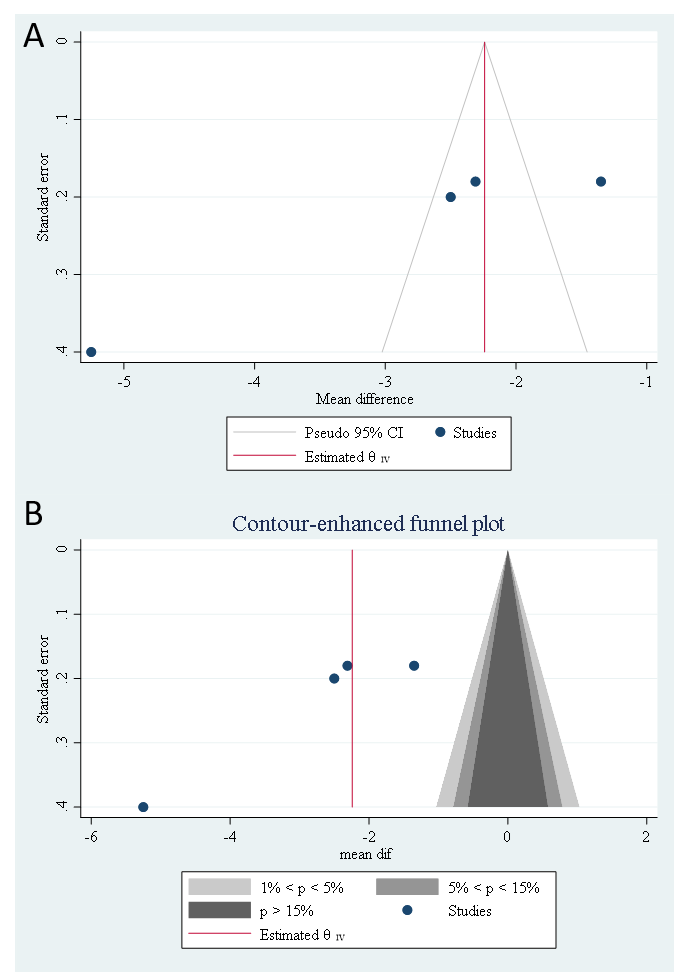
**

**Fig. S3 A** the funnel plot and **B** the contour-enhanced funnel plot for visual analog scale

**
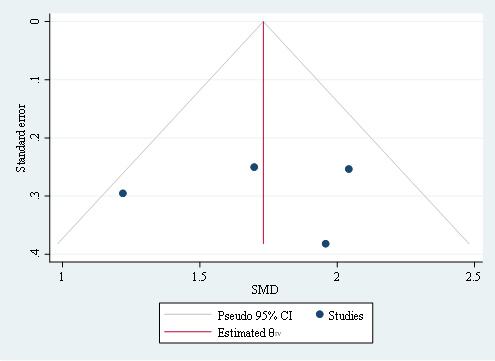
**

**Fig. S4** The funnel plot for functional scores, SMD = standardized mean difference
